# *In vitro* expansion of the mammary stem/progenitor cell population by xanthosine treatment

**DOI:** 10.1186/1471-2121-13-14

**Published:** 2012-06-14

**Authors:** Ratan K Choudhary, Anthony V Capuco

**Affiliations:** 1 Department of Animal and Avian Sciences, University of Maryland, College Park, MD, USA; 2 Bovine Functional Genomics Laboratory, USDA-ARS, Beltsville, MD, USA

**Keywords:** Mammary stem cell, Self renewal, Symmetric division, FNDC3B, Telomerase, Bovine

## Abstract

****Background**:**

Mammary stem cells are critical for growth and maintenance of the mammary gland and therefore are of considerable interest for improving productivity and efficiency of dairy animals. Xanthosine treatment has been demonstrated to promote expansion of putative mammary stem cells *in vivo*, and hepatic and hair follicle stem cells *in vitro*. In the latter, xanthosine promoted the symmetrical division of hepatic and hair follicle stem cells. The objective of this study was to determine if treating primary cultures of bovine mammary epithelial cells (MEC) with xanthosine increases the stem/progenitor cell population by promoting symmetrical division of mammary stem cells.

****Results**:**

*In vitro* treatment with xanthosine increased the population of MEC during the exponential phase of cell growth, reducing the doubling time from 86 h in control cultures to 60 h in xanthosine-treated cultures. The bromodeoxyuridine (BrdU) labeling index and the proportion of MEC in S-phase both were increased by xanthosine treatment, indicating that increased cell accretion was due to increased cell proliferation. Analysis of daughter-pairs indicated that xanthosine promoted a shift from asymmetric to symmetric cell division. Moreover, the 30 % increase in symmetric cell division was concomitant with an increase in the proportion of MEC that were positive for a putative stem cell marker (FNDC3B) and a trend toward increased telomerase activity. These results suggest that xanthosine treatment *in vitro* can increase cell proliferation, promote symmetric cell division and enhance stem/progenitor cell activity.

****Conclusions**:**

Xanthosine treatment increased the proliferation rate of bovine MEC *in vitro*. This was likely to be mediated by an increase in the proportion of stem/progenitor cells in the MEC population due to promotion of symmetrical stem cell division by xanthosine.

## **Background**

Expansion of stem/progenitor cells is prerequisite for their therapeutic or research uses. Mammary stem cells (MaSC) are somatic stem cells that provide for the lineage of mammary epithelial cells. Consequently, they are of considerable interest to developmental biologists, agricultural scientists and cancer researchers. Bovine MaSC have received little attention despite the inherent economic importance of the species and the potential for influencing animal production by MaSC manipulation, and despite the similarity in cytoarchitecture of bovine mammary tissue to that of the human breast [1]. Bovine mammary epithelial cells and their stem cells are important in agriculture production and bioengineering applications. Additionally, information gained will potentially broaden our knowledge of human mammary epithelial cells and stem cells.

Somatic stem cells can divide symmetrically or asymmetrically. Symmetric division of stem cells produces two identical stem cells and results in expansion of the stem cell population. Asymmetric division produces a stem cell and a progenitor cell of more committed cell lineage, while maintaining the existing stem cell population. A novel method of expanding somatic stem cells *in vitro*, based on suppression of asymmetric cell kinetics, was initially identified for rat hepatic stem cells [2] and was recently expanded to include hair follicle stem cells, which renew the epidermis and other components of the skin [3]. Several *in vitro* experiments indicated that p53 promotes asymmetric proliferation of somatic stem cells through suppression of inosine-5’-monophosphate dehydrogenase (IMPDH) [3-6], a rate-limiting enzyme in guanine ribonucleotide biosynthesis. A decrease in guanine ribonucleotide concentrations in these *in vitro* model systems promotes the non-random segregation of sister chromatids that has been shown to be characteristic of asymmetric division of many somatic stem cells. Compounds like xanthosine (a purine nucleoside) bypass IMPDH mediated guanine ribonucleotide synthesis and increase guanine ribonucleotide concentrations in the cell, thereby promoting symmetric division and expanding the stem cell population [2].

Earlier, we reported that xanthosine promotes expansion of label-retaining epithelial cells (LREC), putative stem or progenitor cells, in prepubertal mammary gland *in vivo*[7]. In order to understand the role of xanthosine, we evaluated, in the current study, the growth characteristics of xanthosine-treated cultures of primary bovine mammary epithelial cells. The objective of this study was to investigate the impact of xanthosine on cell proliferation and the kinetics of stem/progenitor cell expansion. We show that xanthosine enhances cell proliferation, promotes symmetrical cell division and provide supportive evidence that it increases the stem/progenitor cell population. Symmetric division was assessed by analyzing the distribution of prelabeled-DNA between pairs of daughter cells and stem cell number was assessed by expression of a potential novel stem cell marker (FNDC3B) and by telomerase activity.

## **Results and Discussion**

### **Growth kinetics and effect of xanthosine**

**Figure 1:**
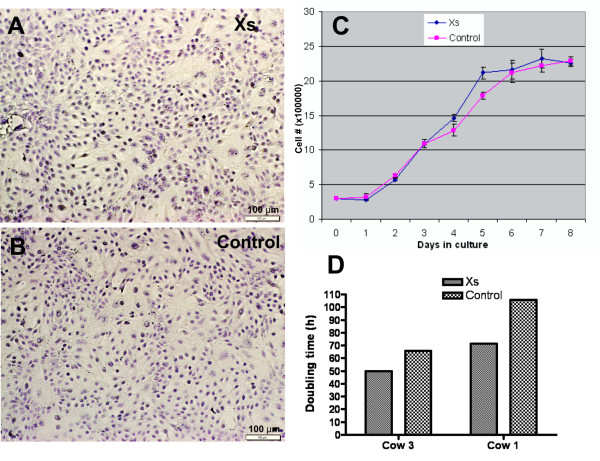
**Evaluation of the effects of xanthosine on cell morphology and growth rate of bovine MEC.** A-B**-** Photomicrographs of hematoxylin stained monolayers of early-passage bovine MEC displaying typical cobblestone morphology of epithelial cells when cultured in xanthosine (**A**) or control (**B**) medium. Scale bar = 100 μm (photographed at 200X). **C**- Growth of primary MEC in the presence and absence of 200 μM xanthosine. Data, from a representative experiment using MEC at passage 3, are presented as the mean number of cells per well (± standard error) for triplicate cultures. An increase (*P* < 0.05) in cell number of xanthosine-treated cultures was evident on days 4 and 5. **D**- Bar graph depicting doubling time of MEC in xanthosine-treated and control cultures using MEC from two cows. Doubling time of xanthosine-treated cultures was consistently lower than that of control cultures regardless of the source of MEC.

The initial experiment evaluated the impact of xanthosine on growth of primary bovine mammary epithelial cells (MEC). MEC plated on plastic growth surfaces in both xanthosine-treated and control cultures displayed a cobblestone-like morphology typical of epithelial cells in monolayer (Figure 1A-B). This cell morphology is consistent with characteristics of bovine mammary epithelial cells as reported by others [8]. The viability assessed by trypan blue staining of xanthosine-treated cells and control cells did not differ (mean >98 %). These observations suggest that neither cell morphology nor cell death rate was influenced by xanthosine treatment. The population growth rate was equivalent for both groups until day 3 of culture but diverged on days 4 – 6 (*P* < 0.05). On days 4 and 5, xanthosine-treated cultures showed an 18 % increase in cell number over control cultures. Cell number plateaued by day 7 for both treatment groups, as the cultures reached confluence (Figure 1C). Doubling time was calculated during the last 3 days of the exponential phase of growth using population doubling time software (http://www.doubling-time.com) [9]. At passage 3, doubling times were 50 h vs. 66 h for xanthosine and control cultures, respectively (Figure 1D). MEC from a different cow at passage 6 grew more slowly and doubling times were 71 h vs. 106 h for xanthosine and control cultures, respectively (Figure 1D). Overall, the doubling time of xanthosine-treated cultures was 71 % of that for control cultures.

### **Cell cycle**

**Figure 2:**
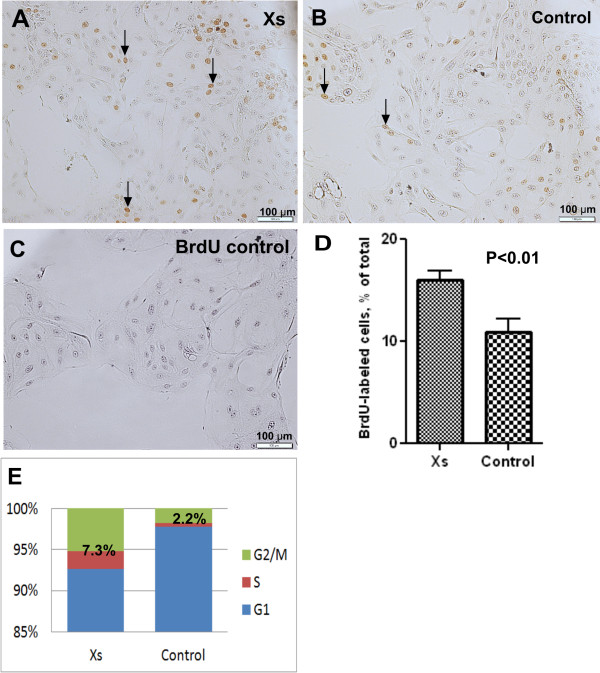
**Effect of xanthosine on cell cycle progression.** A-D- BrdU labeling of proliferating cultures. Photomicrograph depicting BrdU-labeled cells (brown nuclei, arrows) in xanthosine-treated (**A**) and control (**B**) cultures. Negative control for BrdU immunostaining (**C**). Quantification of the BrdU labeling index in xanthosine-treated and control cultures (**D**), expressed as a percentage of total cells. Treatment with xanthosine led to an increase in the percentage (mean + SE, n = 6) of BrdU-positive cells. Scale bar = 100 μm (photographed at 200X). E - Flow cytometry analysis of cell cycle distribution for MEC in xanthosine-treated and control cultures. Xanthosine treatment led to an increase in the number of cells in S + G2/M phases of the cell cycle.

To assess the impact of xanthosine treatment on the proportion of cells in S-phase, cultures were pulse labeled with the thymidine analogue, BrdU. The nuclei of BrdU-labeled cells were clearly evident in both control and xanthosine-treated cultures (Figure 2A-B); but no staining was evident in negative controls for BrdU staining, produced by omission of primary antibody (Figure 1C) or omission of BrdU labeling (not shown). Consistent with the increased population growth rate of xanthosine-treated cells, cultures that were continuously exposed to xanthosine for five days showed an increase (*P* < 0.05) in the proportion of cells in S-phase (16 ± 0.8 % n =6) compared with control cultures (10.8 ± 1.2 %; n =5) (Figure 2D). Additionally, cell cycle analysis of xanthosine-treated and control cultures demonstrated that the percentage of cells in the S-phase plus G2/M-phases was greater in xanthosine-treated than control cultures (7.3 % vs. 2.2 %; Figure 2E). Differences in the proliferation indices between these two experiments are likely a function of the duration of BrdU labeling and the proliferation status of cells in the two experiments presented. Together with the lack of effect of xanthosine on cell viability, these data indicate that xanthosine decreases the culture doubling time by promoting proliferation of MEC.

### **Symmetrical cell division and stem/progenitor cells**

**Figure 3:**
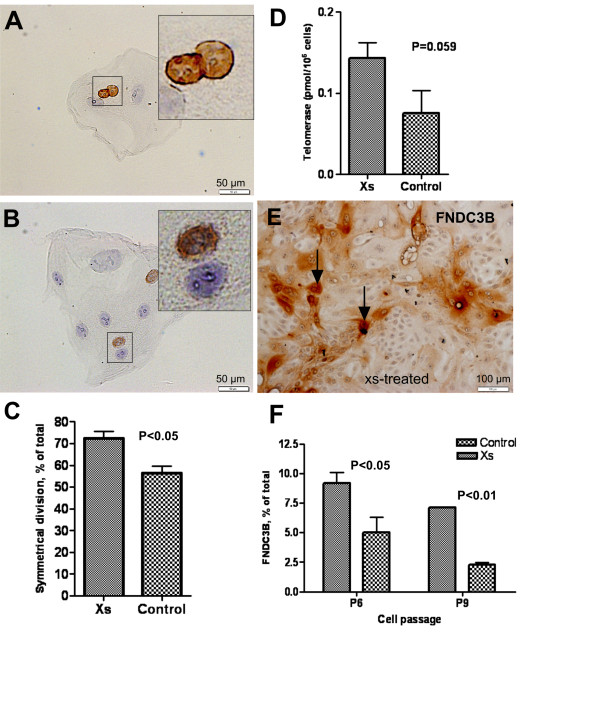
**Detection of symmetric and asymmetric cell division by daughter pair analysis.** Representative photomicrographs illustrating symmetric division (**A**) and asymmetric division (**B**) of MEC (insets are higher magnification images of two daughter cells depicted in the panel). **C-** Treatment of MEC with xanthosine led to an increase in the proportion of cells undergoing symmetric cell division (*P* < 0.05) (representative data of three independent experiments). **D**- Increase in telomerase activity per cells in xanthosine-treated culture in comparison to control culture. A representative photomicrographs of FNDC3B immunostaining in xanthosine-treated (**E**) cultures. **F**- xanthosine led to an increase in the percentage (mean + SE, n = 3) of FNDC3B (+) cells across different passage (P). Scale bars: (A-B) =50 μm (photographed at 320X); (E) =100 μm (photographed at 200X).

To evaluate the influence of xanthosine on symmetric/asymmetric division, an *in situ* immuno-cytochemical assay was used to visualize the distribution of BrdU-labeled DNA between the two daughter cells of a parental cell division. MEC pre-labeled with BrdU, were harvested and seeded at a very low cell density (200 cells/ml/well of 24 well culture plate) and the distribution of label was then tracked in daughter cells of a parental division. When grown in the absence of xanthosine, 56 % of daughter pairs contained two BrdU-positive cells, indicative of symmetrical cell division and the even distribution of BrdU-labeled DNA between the daughter cells (Figure 3A); whereas 44 % of daughter pairs contained a single BrdU-positive cell, indicative asymmetrical parental cell division (Figure 3B). In contrast, when cells were incubated with xanthosine, the proportion of cells undergoing symmetrical cell division increased from the 56 % observed in control cultures to 72 % in xanthosine-treated cultures (*P* < 0.05; Figure 3C). We assume that these dividing cells contain a substantial proportion of stem cells (or progenitor cells) and that their population is expanded by the symmetrical division induced by xanthosine. This is consistent with *in vitro* studies by Sherley and colleagues, who demonstrated that *in vitro* treatment of cells with xanthosine increased symmetrical division of hepatocyte [2] and hair follicle stem cells [3].

Additional support for the conclusion that xanthosine increased the population of stem/progenitor cells is provided by increased telomerase activity and FNDC3B expression by xanthosine-treated cells. We observed that xanthosine-treated cultures tended to have more telomerase activity (pmol/10^6^ cells) than control cultures (0.14 vs. 0.075; *P* < 0.06) (Figure 3D). This trend toward increased telomerase activity in xanthosine-treated cells was consistently observed in repeated experiments. Because telomerase activity is primarily found in stem cells and progenitor cells [10], these data are consistent with a xanthosine-induced increase in the population of these cells. Furthermore, we showed that xanthosine treatment increased the number of FNDC3B-positive cells in culture (Figure 3E, arrows). FNDC3B is a potential marker of bovine mammary stem and progenitor cells that we recently identified by microarray analysis of LREC (data deposited in NCBI Gene Expression Omnibus; http://www.ncbi.nlm.nih.gov/geo/query/acc.cgi?acc=GSE31541), which were excised by laser microdissection from cryosections of mammary tissue [11]. We quantified the percentage of FNDC3B-positive cells at passages 6 and 9, and found an increased percentage (*P* = 0.015) of FNDC3B-positive cells (Figure 3E) in xanthosine-treated cultures compared with control cultures (9.2 ± 0.9 % vs. 5.0 ± 1.2 % for passage 6, and 7.1 ± 1.3 % vs. 2.2 ± 0.4 % for passage 9; Figure 3F). The decline (*P* < 0.01) in the percentage of FNDC3B-positive cells (9.2 % to 7.1 %) from passage 6 to passage 9, was abrogated by treatment with xanthosine. We believe that our data generated using this putative MaSC marker has provided the first quantitative evidence in support of the ability of xanthosine to limit the predicted decline in the population of stem cells that was postulated to occur as a result of dilution caused by the preponderance of asymmetric cell kinetics during normal cell culture and passage [12].

This *in vitro* study strengthens the hypothesis that xanthosine treatment increases the population of bovine mammary stem/progenitor cells*.* These results are consistent with an earlier *in vivo* study of prepubertal bovine mammary gland, wherein intramammary infusion of xanthosine increased the population of putative MaSC, which were identified on the basis of long-term retention of BrdU-label [7], The results are also consistent with a recent *in vivo* study. At the author’s suggestion, colleagues utilized a protocol designed to expand the MaSC population of transgenic goats by intramammary infusion of inosine, which, like xanthosine, is a guanine nucleotide precursor [13]. The treatment resulted in a 62 % increase in milk production, which emphasizes the potential benefits that may be garnered by *in vivo* modulation of stem cell function.

Because MaSC provide for growth and renewal of the mammary epithelium, the ability to modulate their number provides a means to modulate tissue growth, repair and cell turnover, which in turn can significantly influence animal productivity. In face of an ever-increasing world population, a beneficial effect on animal productivity should not be underappreciated.

## **Conclusions**

In the present study, we evaluated the impact of xanthosine on proliferation of bovine MEC in culture. We found that xanthosine enhanced proliferation of bovine MEC. Xanthosine increased the number of stem/progenitor cells in culture, as evidenced by an increase in the expression of FNDC3B and telomerase activity, and that this was accomplished by enhancing symmetrical division of mammary stem cells. This study provides support for the ability of xanthosine to expand the mammary stem cell population and support for the exciting prospect of regulating mammary stem cell number by treatment with a naturally occurring nucleoside.

## **Methods**

### **Cultivation of bovine primary mammary epithelial cells**

Frozen stocks of bovine mammary epithelial (MEC) cells from non-lactating cows were kindly provided by Dr. David Kerr, University of Vermont. Isolation of these cells was described previously [14]. Briefly, mammary tissues were obtained from pregnant, mastitis-free donor cows (6–9 months pre-partum) at the time of sacrifice. Tissues were minced into small pieces, rinsed in Hanks Balanced Salt Solution (HBSS) and then subjected to enzymatic digestion (collagenase, hyaluronidase, and DNAse I) in HBSS supplemented with antibiotics and anti-fungal agents. After a 3 h digestion (37 °C), cells were filtered through meshes of decreasing pore size (~1 mm^2^ to 100 μm^2^). Cell pellets were washed, centrifuged and resuspended in medium (DMEM/F12 with 3 % fetal bovine serum and penicillin/streptomycin) and plated in plastic culture flasks to allow fibroblasts to attach to the substratum. After 30 min at 37 °C, cells were decanted and used as a source of primary mammary epithelial cells. Cells were passaged twice and then cryopreserved in 1 ml aliquots containing approximately 2 × 10^6^ cells in DMEM/F12 containing 20 % FBS and 10 % dimethylsulfoxide.

### **Cell culture conditions**

Frozen cells were revived in growth medium consisting of DMEM/F12 with 5 % FBSITS supplement (5 μg/ml insulin, 5 μg/ml transferrin and 0.005 μg/ml sodium selenite), glutamine dipeptide (2 mM; GlutaMAX™-I), penicillin G (100 μg/ml), and streptomycin (100 μg/ml) and plated in a 75-cm^2^ tissue culture flask (T75, Corning Inc., Corning, NY, USA). These medium supplements were obtained from Gibco/Invitrogen (Carlsbad, CA, USA). Initially, cells were allowed to attach to the bottom of the T75 tissue culture flask for 5 h and then the medium was replaced with fresh medium. Cells were grown at 37 °C in a humidified atmosphere of 5 % CO_2_ in air. For cell passage, the medium was discarded and the monolayer washed with phosphate buffer saline (PBS). Cells were then incubated in 2 ml of 0.05 % trypsin-EDTA (Invitrogen, Carlsbad, CA) for 10–15 min at 37 °C, until most cells became detached. Trypsinization was stopped by adding 5 ml of growth medium. After centrifugation (23 °C, 6 min, 210 × *g*), the cell pellet was resuspended in 5 ml of growth medium. For routine passage, cells were split at a 1:4 ratio. Cells grew to 70-80 % confluence in 5 to 7 days, at which time they were subcultured. Medium was replaced every 2–3 days.

Two populations of MEC, isolated from two different animals, were used in this study. Evaluation of the impact of xanthosine on culture dynamics (cell number, population doubling time, viability and morphology) and on telomerase activity utilized both populations at multiple passages. Subsequent experiments were conducted using the population of cells that grew faster (facilitating experimental replication).

### **Growth characteristics of bovine mammary epithelial cells**

To evaluate the impact of xanthosine on growth characteristics of MEC, cells were seeded at a density of 3 × 10^4^ cells/well of 24-well flat bottom tissue culture plates (Falcon, Oxnard, CA, USA) containing one ml of growth medium with or without 200 μM xanthosine (Sigma, Saint Louis, MO), prepared from a 10 mM stock solution of xanthosine (prepared in alkaline distilled water and filtered through a sterile membrane, 0.2 μ pore size). Cell number and viability of triplicate wells were determined daily for 8 days. Cell number was assessed by haemocytometer counts of trypsinzed cell suspensions and viability by the dye exclusion, using 0.4 % trypan blue (Gibco, Carlsbad, CA, USA). Morphology of cells in confluent monolayers was evaluated by microscopy on day 8 after hematoxylin staining. Three independent experiments were conducted using two populations of MEC, each isolated from a different cow.

### **Bromodeoxyuridine labeling index and FNDC3B staining**

MEC were seeded in at a density of 6 x 10^5^ cells in 5-cm^2^ Petri dishes (Corning) containing 2 ml of growth medium with or without 200 μM xanthosine and grown for five days (~80 % confluent). Bromodeoxyuridine (BrdU; Sigma, St Louis, MO, USA) was added to culture medium at a concentration of 10 μM during the logarithmic phase of growth. Cells in six replicate plates per treatment were incubated with BrdU for 5 h, after which the monolayers were washed with chilled PBS (2 × 1 min) and fixed in pre-chilled methanol for 15 min at −20 °C. Cells were then treated with 0.3 % Triton X-100 (Sigma) for 30 min, and endogenous peroxidase was blocked with Peroxo-block (Invitrogen) for 1 min. Cells were treated with 2 N HCl for 30 min at room temperature (RT) followed by acid neutralization with 0.1 M borate buffer (2 x 5 min) and 3 washes (3 × 2 min) in PBST (PBS + 0.05 % triton X-100). Cells were blocked with casein (CAS-Block^TM^, Invitrogen) for 10 min, and then incubated with mouse monoclonal anti-BrdU (Roche Diagnostics, Mannheim, Germany) at 1:100 dilution in CAS-Block for 1–2 h at RT. After washing with PBST (3 x 3 min) cell monolayers were incubated with Vector ImmPRESS™ anti-mouse/anti-rabbit Ig peroxidase conjugated polymer detection reagent (Vector Labs Inc., Burlingame, CA, USA) for 30 min, followed by washing with PBST (3 x 2 min) to remove unbound polymer. Immunostained cells were visualized with DAB (Vector). Negative control staining was performed by omitting primary antibody. Sections were counter-stained with hematoxylin for 1 min, washed briefly in water and color developed in PBS (30 s). Cells were dehydrated in ascending concentrations of ethanol. Photomicrographs were obtained using brightfield optics with an Olympus BX81 microscope (Olympus, Japan) equipped with a DP70 digital camera. The percentage of immuno-positive cells was enumerated from photomicrographs of 10–12 random fields per culture plate.

FNDC3B staining was performed as BrdU staining, except that neither HCl treatment nor other antigen retrieval step was used prior to incubation with primary antibody. Primary antibody was a rabbit polyclonal (Santa Cruz Biotechnology, Santa Cruz, CA, USA) used at 1:100 dilution. The influence of xanthosine and culture passage on the FNDC3B labeling index was tested by two-way ANOVA with Bonferroni correction using GraphPad Prism (version 3; GraphPad Software Inc., San Diego, CA, USA).

### **Flow cytometry analysis**

To support evaluations of population doubling time and BrdU labeling index, we analyzed the impact of xanthosine on cell cycle distribution, using a flow cytometry assay. BrdU (10 μM) was added to cell culture medium for 45 min during the log phase of cell growth. Cells were then harvested by trypsinization and fixed with 70 % ethanol in PBS overnight at 4 °C. Cells were pelleted, permeabilized with 0.3 % triton-X100 in PBS for 30 min at RT, and then re-pelleted. Nuclei were suspended and incubated in 2 N HCl for 20 min at 37 °C and then neutralized with 0.1 M sodium borate buffer. Nuclei were pelleted and re-suspended in PBST before blocking with 200 μl of casein (CAS Block, Invitrogen) for 10 min and incubating with an Alexa 488-conjugated BrdU antibody (4 μg/ml, Molecular Probes, Invitrogen) at RT for 40 min in the dark. Samples were then rinsed twice in PBST and resuspended in 500 μl of propidium iodide/RNase staining buffer (BD Pharmingen, San Diego, CA) for 15 min before flow cytometry. Samples were kept on ice in the dark before analysis. Analyses were performed on a FC500 flow cytometer (Beckman Coulter Inc., Palatine, IL) and collected data were analyzed using Cytomics RXP (Beckman). This experiment was repeated twice with 3–5 replicates per group, using cells at two different passages.

### **Segregation of labeled DNA in daughter cells**

The distribution of prelabeled-DNA was tracked in daughter cells using an approach analogous to that employed by Conboy et al. to evaluate template strand segregation of muscle stem cells *in vivo*[15]. Cells were seeded at 1.5 × 10^5^ per T25 flask and grown until approximately 80 % confluent. Incorporation of BrdU into nuclear DNA was performed during the log phase of growth to label maximum number of cycling cells. BrdU (10 μM) was added to the medium for 1 h prior to cell harvest. After trypsinization, cells were plated at a concentration of 200 cells per well (n = 4) of a 24-well plate. This cell density allowed for discrimination of individual cells and subsequent daughter-pairs. The influence of xanthosine on the proportion of cells undergoing symmetric vs. asymmetric division was evaluated by seeding cells in control medium or medium containing 200 μM of xanthosine. Cells were cultured for an additional 48 h (doubling time 50–60 h). The cell cultures were then washed with chilled PBS (2 × 1 min), fixed in methanol at −20 °C and processed for BrdU immunocytochemistry. Cell pairs containing one or two BrdU-labeled cells were counted to determine the percentage of asymmetrical (one labeled daughter cell) versus symmetrical (two labeled daughter cells) division. Three independent experiments were performed and provided analogous results. Results of a single representative experiment are presented.

### **Telomerase assay**

To determine cellular telomerase activity, MEC were seeded in 12-well tissue culture plates (Corning) at 6 × 10^4^ cells per well in 2 ml of media with or without 200 μM of xanthosine. At 80 % confluence, 0.2 ml of trypsin-EDTA solution was added to each well and incubated for 15 min at 37 °C. Cells were dislodged and a single cell suspension obtained by titurating in the micropipette tip. Then 0.8 ml of growth medium was added to inhibit trypsin activity and the cell suspension collected in a 1.5 ml microcentrifuge tube. A 50 μl aliquot of the cell suspension was removed from each tube to assess cell viability and cell number. The remaining cell suspension (950 μl) was used to determine telomerase activity using a quantitative real-time PCR-based telomerase assay (US Biomax, Rockville, MD, USA). Briefly, the cell suspension was centrifuged at 220 x g for 6 min and the pelleted cells (~ 1.0 - 1.5 × 10^5^ cells) were lysed by vortexing for 1 min in 200 μl of the provided lysis buffer and incubating on ice for 30 min. The cell lysate was then centrifuged for 30 min at 12,000 x g at 4 °C. The supernate was removed and collected in a 0.5 ml plastic microfuge tube. A portion of each supernate was heat inactivated (85 °C for 10 min) and served as the negative control for that sample. One μl of each of sample supernate and heat-inactivated supernate was run in the assay along with a serial dilution of the provided standards in the lysis buffer, according to the manufacturer’s recommendations.

Mention of trade names or commercial products in this article is solely for the purpose of providing specific information and does not imply recommendation or endorsement by the US Department of Agriculture. The USDA is an equal opportunity provider and employer.

## **Competing interests**

The authors declare that they have no competing interests.

## **Authors’ contributions**

AVC and RKC established the hypotheses and planned the experiments. RKC performed the experiments and drafted the manuscript. AVC finalized the manuscript. Both authors read and approved the final manuscript.

## **Authors’ information**

AVC is a Research Physiologist (Animal) at Bovine Functional Genomics Laboratory, USDA, Beltsville and an Adjunct Professor at the University of Maryland, College Park, Maryland, USA.

RKC is currently a postdoctoral fellow in the Department of Animal and Food Sciences, University of Kentucky. He is located in the Bovine Functional Genomics Laboratory, USDA-ARS, Beltsville, Maryland, USA.
